# Supplementary material for: Usefulness of several factors and clinical scoring models in preoperative diagnosis of complicated appendicitis
Source: PLoS One. 2021 Jul 27;16(7):e0255253. doi: 10.1371/journal.pone.0255253 (PMC8315522; doi:10.1371/journal.pone.0255253)
Supplement: Attachment — Submitted filename: Response_to_Reviewers_KFujiwara.docx [file pone.0255253.s002.docx]

Sada Hospitall

2-4-28 Watanabe Street

Chuo-Ward, Fukuoka 810-0004 JAPAN

+81-92-781-6381 Tel/Fax

kengdom@surg1.med.kyushu-u.ac.jp

https://www.linkedin.com/in/kenjifujiwara/?locale=en_US

**Kenji Fujiwara, M.D., Ph.D.**

*General surgeon, Department of Surgery,*

*Sada Hospital*

PONE-D-21-12222

**Usefulness of several factors and clinical scoring models in preoperative diagnosis of complicated appendicitis**

*PLOS ONE*

June 29, 2021

Dear Editors and Reviewers,

We are very pleased to re-submit our manuscript, entitled “Usefulness of several factors and clinical scoring models in preoperative diagnosis of complicated appendicitis” by Fujiwara et al. for your consideration for publication in *PLOS ONE*.

Thank you for taking the time to review our revised manuscript. We believe our manuscript is now fit for the criteria for the journal. Below, we have listed each comment, along with our subsequent revisions and responses.

Sincerely,

Kenji Fujiwara, M.D., Ph.D.

**Reviewer #1:** For diagnosing acute appendicitis, both clinical and laboratory findings individually do not suffice, combined with standard imaging increases the diagnostic power for acute appendicitis. Incorporating imaging features in clinical scoring models may provide better differentiation between uncomplicated and complicated appendicitis. Optimizing patient selection for treatment of appendicitis resulting in better treatment outcomes. To emphasize the CRP value may misdiagnose with other abdominal diseases.

Author Response: Thank you for the thoughtful suggestion. We agreed that we exaggerated the importance of CRP too much, and this may mislead the readers. We arranged some sentences to indicate that CRP may be useful when supported by clinical findings and imaging.

We summarized the changes below.

1. We added the sentence “when supported by clinical findings and imaging” in the abstract (Page 3): Specifically, high serum levels of CRP may be a useful factor in predicting complicated appendicitis prior to surgery **when supported by clinical findings and imaging;** however, further research is needed.
2. We deleted the sentences “Great benefit of standard blood draws is easier and less invasive than imaging like CT scan (Page 16; Line 240-241)” because this sentence may mislead readers to think that CRP can substitute the imaging.
3. We added the sentence “when supported by clinical findings and imaging” in conclusion (Page 17): We propose that CRP may be a useful factor in predicting complicated appendicitis **when supported by clinical findings and imaging**, and look forward to further research of this factor.

**Reviewer #3:** I have read with interest this submission. The authors have for the most part revised and addressed the recommendations of the very thorough review performed by the 1st round of reviewers. As I read the manuscript, I have only identified a minor issue which I think need to be addressed.
As it is known, CRP is a nonspecific acute phase reactant and may increase for many reasons. It would be a overestimated statement to say that CRP alone is a useful factor in predicting complicated appendicitis. In the discussion, it would be appropriate to edit the statement as "may be useful when supported by clinical findings and imaging".

Author Response: Thank you for the thoughtful suggestion. We agreed that we exaggerated the importance of CRP too much, and this may mislead the readers. We arranged some sentences to indicate that CRP may be useful when supported by clinical findings and imaging.

We summarized the changes below.

1. We added the sentence “when supported by clinical findings and imaging” in the abstract (Page 3): Specifically, high serum levels of CRP may be a useful factor in predicting complicated appendicitis prior to surgery **when supported by clinical findings and imaging;** however, further research is needed.
2. We deleted the sentences “Great benefit of standard blood draws is easier and less invasive than imaging like CT scan (Page 16; Line 240-241)” because this sentence may mislead readers to think that CRP can substitute the imaging.
3. We added the sentence “when supported by clinical findings and imaging” in conclusion (Page 17): We propose that CRP may be a useful factor in predicting complicated appendicitis **when supported by clinical findings and imaging**, and look forward to further research of this factor.
